# A Systematic Review on the Genetic Contribution to Tinnitus

**DOI:** 10.1007/s10162-024-00925-6

**Published:** 2024-02-09

**Authors:** Patricia Perez-Carpena, Jose A. Lopez-Escamez, Álvaro Gallego-Martinez

**Affiliations:** 1 Otology and Neurotology Group CTS495, Division of Otolaryngology, Department of Surgery, Instituto de Investigación Biosanitaria, Ibs.GRANADA, Universidad de Granada, Granada, Spain; 2 Sensorineural Pathology Programme, Centro de Investigación Biomédica en Red en Enfermedades Raras, CIBERER, Madrid, Spain; 3 Department of Otolaryngology, Instituto de Investigación Biosanitaria Ibs.GRANADA, Hospital Universitario Virgen de Las Nieves, Granada, Spain; 4 Meniere’s Disease Neuroscience Research Program, Faculty of Medicine & Health, School of Medical Sciences, The Kolling Institute, University of Sydney, Sydney, NSW Australia

**Keywords:** Tinnitus, Heritability, Genetics, GWAS, Prevalence, Rare variation

## Abstract

**Purpose:**

To assess the available evidence to support a genetic contribution and define the role of common and rare variants in tinnitus.

**Methods:**

After a systematic search and quality assessment, 31 records including 383,063 patients were selected (14 epidemiological studies and 17 genetic association studies). General information on the sample size, age, sex, tinnitus prevalence, severe tinnitus distribution, and sensorineural hearing loss was retrieved. Studies that did not include data on hearing assessment were excluded. Relative frequencies were used for qualitative variables to compare different studies and to obtain average values. Genetic variants and genes were listed and clustered according to their potential role in tinnitus development.

**Results:**

The average prevalence of tinnitus estimated from population-based studies was 26.3% for any tinnitus, and 20% of patients with tinnitus reported it as an annoying symptom. One study has reported population-specific differences in the prevalence of tinnitus, the white ancestry being the population with a higher prevalence. Genome-wide association studies have identified and replicated two common variants in the Chinese population (rs2846071; rs4149577) in the intron of *TNFRSF1A*, associated with noise-induced tinnitus. Moreover, gene burden analyses in sequencing data from Spanish and Swede patients with severe tinnitus have identified and replicated *ANK2*, *AKAP9*, and *TSC2* genes.

**Conclusions:**

The genetic contribution to tinnitus is starting to be revealed and it shows population-specific effects in European and Asian populations. The common allelic variants associated with tinnitus that showed replication are associated with noise-induced tinnitus. Although severe tinnitus has been associated with rare variants with large effect, their role on hearing or hyperacusis has not been established.

**Supplementary Information:**

The online version contains supplementary material available at 10.1007/s10162-024-00925-6.

## Introduction

Tinnitus has been considered an annoying symptom usually associated with sensorineural hearing loss (SNHL) or anxiety commonly found in the aging population [1]; its origin is multifactorial, but it is attributed mostly to environmental factors, noise exposure being the best-known risk factor [2]. Although tinnitus prevalence studies across different populations are scarce, multiple epidemiological studies in large cohorts of individuals with tinnitus, including twins [3, 4], adoptees [5], and familial aggregation studies [6] have reported evidence to support a significant tinnitus heritability, particularly for severe bilateral tinnitus [3, 7]. This hidden inheritance is explained by genetic variation in the DNA sequence and different genetic studies have reported common and rare variants associated with different tinnitus phenotypes [7–11].

A precise phenotype definition is an essential requisite for genetic association studies in complex traits, since the effect size of common and rare variants on the phenotype is small or large according to their allelic frequency [12]. For rare variants, the effect can be large, small, or negligible, but for common variants, the effect only can be small or moderate. As a result, the frequency of the tinnitus phenotype is related to the expected additive effect of common and rare variants, determining the effect size associated with the phenotype [13, 14].

The term “tinnitus disorder” has been proposed for a rare form of tinnitus reported in around 1–2% of the population that is associated with emotional distress, cognitive dysfunction, and/or autonomic arousal, leading to behavioral changes and functional disability [15]. These patients also report SNHL and hyperacusis [16] and should be considered a severe form of tinnitus requiring a multidisciplinary intervention for its management [17–19].

The aim of this systematic review is to assess the available evidence to support a genetic contribution and define the role of common and rare variants in the human genome to tinnitus.

## Methods

This review has followed the guidelines from “Preferred Reported Items for Systematic Reviews and Meta-Analyses” [20] (Annex 1). The protocol was registered on PROSPERO (registration number: CRD42023440491).

### Study Design

According to the methodology for systematic reviews, the items related to the PICO question are listed as follows, so the studies have been selected according to the following characteristics:

- Participants: patients with a diagnosis for tinnitus, or referring it as a relevant symptom.
- Intervention: determination of the prevalence of tinnitus, estimation of familial aggregation of this symptom, and measurement of concordance rate between twins. Description of genetic variants potentially linked to the development of chronic tinnitus
- Controls: controlled and uncontrolled studies
- Main outcomes: tinnitus prevalence across different populations, according to the ethnicity and genetic variants associated with tinnitus.
- Study designs: case–control studies, population-based studies, family aggregation studies, and twin studies.

### Search Strategy

The bibliographic search was conducted on 15 May 2023. PubMed and Scopus databases were used with the following combination of MesH terms: (“tinnitus”) AND (“prevalence” OR “inheritance” OR “heritage” OR “heritability” OR “genes” OR “genetics” OR “families” OR “familial” OR “twins”), and it was limited to original articles published in the last 10 years.

**Fig. 1:**
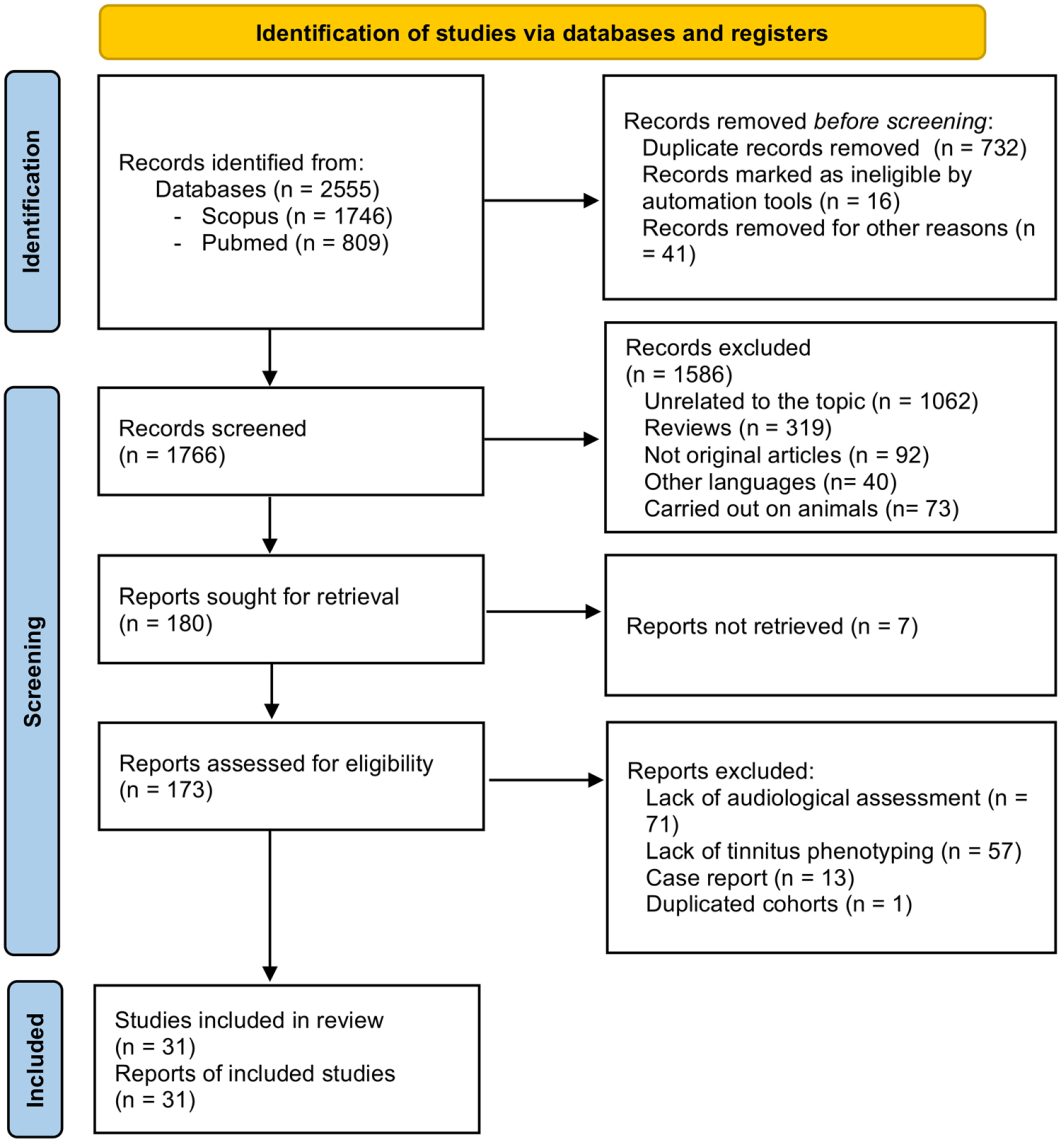
Flow diagram for study selection

Once the search was performed in both databases, duplicates were eliminated and articles whose title or abstract did not adjust to the objectives of the review were discarded. The selected records were read and those that did not meet the inclusion criteria were excluded. The flowchart with the steps followed in this search is shown in Fig. 1.

### Exclusion Criteria

- Studies without any audiological information (pure tone audiometry, auditory brain responses, self-report hearing loss…)
- Studies with self-reported tinnitus without additional phenotyping by psychometric questionnaires
- Animal studies
- Studies published in other languages than English or Spanish
- Single case reports

### Data Extraction

Two reviewers (PP-C, AGM) independently extracted study characteristics and outcomes from all the included studies, and data were compared. A third reviewer (JALE) was consulted when a consensus could not be reached.

Each article was reviewed to extract the most relevant information according to the objective of this review. For population-based studies, information was collected on authors, year of publication, country and continent, target population, years of registry, sample size, and tinnitus prevalence for each study. Information on severe/bothersome tinnitus was retrieved for each record, according to open questions to patients, or quality-of-life questionnaires. In the familial aggregation studies, the information retrieved was as follows: authors and year of publication, sample size, number of relatives with chronic tinnitus, and the total number of siblings in each family. To sum up, in the genetic studies, we include the reference, country, study design, objective, sample size, sex, mean age, and main results of each study.

### Data Synthesis

General information on the sample size, age, sex, tinnitus prevalence, severe tinnitus distribution, and SNHL was recorded. Qualitative variables are presented as relative frequencies to compare them among studies and to obtain average values. Quantitative variables are expressed as mean ± standard deviations (SD). All statistical analyses were performed using SPSS Statistics package v22 (IBM, Armonk, NY).

### Quality Assessment

The quality of each study was analyzed according to the type of study. The Risk Of Bias In Non-randomized Studies—of Exposure (ROBINS-E) tool [21] was used for observational epidemiology studies (prevalence studies) and candidate gene studies. GWAS studies were not assessed through the risk of bias, as no proper tool is available for such aim. The seven domains in this tool include the following: (1) bias due to confounding, (2) bias in the measurement of the exposure, (3) bias in the selection of participants into the study, (4) bias due to post-exposure interventions, (5) bias due to missing data, (6) bias in the measurement of outcomes, and (7) bias in the selection of the reported results. However, domain 4 was not relevant in this review and was excluded. The risk of bias ranged from “Low” to “Moderate,” “High,” or “Very High,” and the overall risk of bias was established based on the judgement for all the domains. A color scale was used to summarize it (green, low risk; yellow, moderate risk; red, high risk; black, very high risk). This has been summarized in Table 3.

## Results

According to the eligibility criteria, 31 studies (383,063 patients) were included (14 prevalence studies and 17 genetic studies). The following flowchart details the selection process and filtering of the included articles (Fig. 1).

### Prevalence Studies for Tinnitus

Fourteen studies were selected to estimate the prevalence of tinnitus, including 88,920 subjects. Three were performed in North America, two of them in the USA [22, 23] and one in Canada [24], and eight were performed in Europe, including studies from Poland [25, 26], UK [27], Netherlands [28], Sweden [4], Germany [29, 30], and Denmark [31], and three in Asia (South Korea) [32–34]. The age ranged from 11 to 99 years, with 46.3 ± 15.2 years old.

The mean prevalence for hearing loss was 25% for all the population-based studies, and 34.7% in the subgroup of patients with self-reported tinnitus, based on pure tone audiograms. The hearing thresholds were 15.6 ± 10 dB HL (pure tone average, PTA) in subjects without tinnitus and 24.4 ± 19.3 dB HL in patients with tinnitus, respectively.

In terms of self-reported tinnitus, the mean prevalence from all the population-based studies was 26.9%, and 20.7% of patients with tinnitus described it as an annoying symptom, based on different phenotyping tools, such as THI, TFI, or a direct question about it burden level.

Next, we compared the prevalence of tinnitus in European, North American, and Asian studies, according to the ethnic background. European studies registered a mean prevalence of tinnitus of 37.3%, with a mean prevalence of 44.2% for SNHL and a mean age of 35.4 ± 26.4 years old. Studies based on the North American population recorded a mean prevalence of tinnitus of 19.1%, with a mean prevalence of 29.2% for SNHL and a mean age of 42.8 ± 1.2 years, and studies in the Asian population a mean prevalence of 20%, with a mean prevalence of 12.3% for SNHL and a mean age of 53.5 ± 3.7 years (Table 1). Two studies analyzed African American populations with a prevalence of 21.6% (mean age 53.7 ± 11.5 years old.

**Table 1:** Summary of the main findings in tinnitus prevalence studies

**Reference**	**Country**	**Study population**	**Period of registration**	**Sample size**	**Average age in years (SD)**	**Tinnitus prevalence**	**Severe tinnitus**	**Hearing loss prevalence**	**Mean PTA values (dB HL)**
Bogo et al. [4]	Sweden	Male mono- and dizygotic twin cohort	1991–1995	1084	49.5 (34–78)	13.5%	2.8%	N/A	No tinnitus: 8.48 dB Tinnitus: 14.15 dB
Dziendziel et al. [25]	Poland	Otosclerosis cohort	2017	460	48 (± 11.5)	64.8%	22.8%	66%*	56.5 dB (AC); 26.7 dB (BC)
Hackenberg et al. [29]	Germany	Population-based cohort study	2017–2020	8539	60.7 (± 13.7)	28%	40.4%^e^	63.6%^e^	N/A
							41%^f^	48.7%^f^	
							41.6%^g^	51%^g^	
Hackenberg et al. [30]	Germany	Population-based cohort study	2017–2020	4942	61 (± 13.3)	26.1%	37.6%	N/A	No tinnitus: 17.17 dB Tinnitus: 22.67 dB
Humphriss et al. [27]	UK	11-year-old children cohort study	2013	7092	11	17.5%	1.9%	N/A	No tinnitus: 3.63 dB Tinnitus: 5.54 dB
Lima et al. [26]	Poland	Otosclerosis cohort	2019	66	48.7 (± 9.1)	72.7%	33.3%	N/A	49.4 ± 12.1 dB (AC); 23.1 ± 8.2 dB (BC)
Nemholt et al. [31]	Denmark	Longitudinal school-based cohort study	2014	501	13.7 (± 1.1)	54.3%	34.6%	8%	N/A
Oosterloo et al. [28]	Netherlands	Population-based cohort study	2011–2016	6098	61.8 (± 11.5)	21.4%	12.3%	29.2% (no tinnitus) 43.2% (tinnitus)	No tinnitus: 29.1 dB (± 16.5) Tinnitus: 35.4 dB (± 19.2)
Park et al. [33]	South Korea	Population-based cohort study	2009–2011	21,893	57.23^ h^ 53.68^i^	19.7%	29.3%	15.2%	N/A
Park et al. [34]	South Korea	Population-based cohort study	2009–2011	10,061	49.7 (± 15.8)	21.36%	34.24%	15.2%	N/A
Choi et al. [32]	South Korea	Population-based cohort study	2010–2012	16,630	N/A (12–99)	20% (normal hearing) 18.8% (SNHL)	5.65%	6.4%	N/A
Choi et al. [22]	USA	Population-based cross-sectional study	2011–2012	3669^a^	43.8 (± 14.4)^a^	16.5%^a^	6.6%^a^	8–26.1%^a^	N/A
				530^b^	41.4 (± 13.9)^b^	6.6%^b^	2.4%^b^	6.2–21.5%^b^	
				1329^c^	43.2 (± 13.9)^c^	18.8%^c^	5.7%^c^	9.9–28%^c^	
				1047^d^	45.5 (± 14.9)^d^	13.6%^d^	4%^d^	6.7–24.5%^d^	
				763^e^	40 (± 14.4)^e^	11.9%^e^	16.7%^e^	7.8–27.9%^e^	
House et al. [23]	USA	African-American cohort study	2008–2014	1314	61.8 (± 11.5)	29.5%	35.4%	N/A	No tinnitus: 19.4 dB (± 10.2) Tinnitus: 23.4 dB (± 11.8)
Ramage-Morin et al. [24]	Canada	Population-based cohort study	2012–2015	6571	NA (19–79)	36.6%	6.5%	23.5% (SNHL) 14.5% (tinnitus + SNHL)	N/A

*AC* air conduction, *BC* bone conduction, *PTA* pure tone average, *SD* standard deviation, *SNHL* sensorineural hearing loss

^a^Study cohort

^b^Asian American

^c^White

^d^Black

^e^Hispanic

^f^European standard population 2013

^g^German standard population 2021

^h^mean age for the tinnitus group

^i^mean age for the non-tinnitus group

*conductive/mixed hearing loss

### Genetic Studies

Seventeen genetic studies were included [8, 9, 35–49], with a global sample size of 294,143 subjects (45.4% males), and 55,267 patients with tinnitus (18.8% males).

All the studies included reported no significant differences in terms of age between subgroups. Three studies excluded patients with SNHL [36, 42, 47], two studies reported no significant differences in audiograms between tinnitus and control subjects [39, 40], six studies included subjects with some degree of hearing impairment, including SNHL [8, 37, 45, 49], presbycusis [35], or noise-induced hearing loss [41], and the rest of them failed to report information on hearing stage [9, 38, 43, 44, 46, 48].

These genetic studies included four genome-wide association studies (GWAS) using genotyping arrays [9, 41, 46, 47], nine candidate gene studies [35, 36, 38–40, 42, 43, 45, 48], two sequencing studies using exome sequencing [8] and genome-sequencing data [49], and one mitochondrial DNA sequencing study [37]. Besides, there was a methylation study in the *BDNF* and *GDNF* genes [44].

**Fig. 2:**
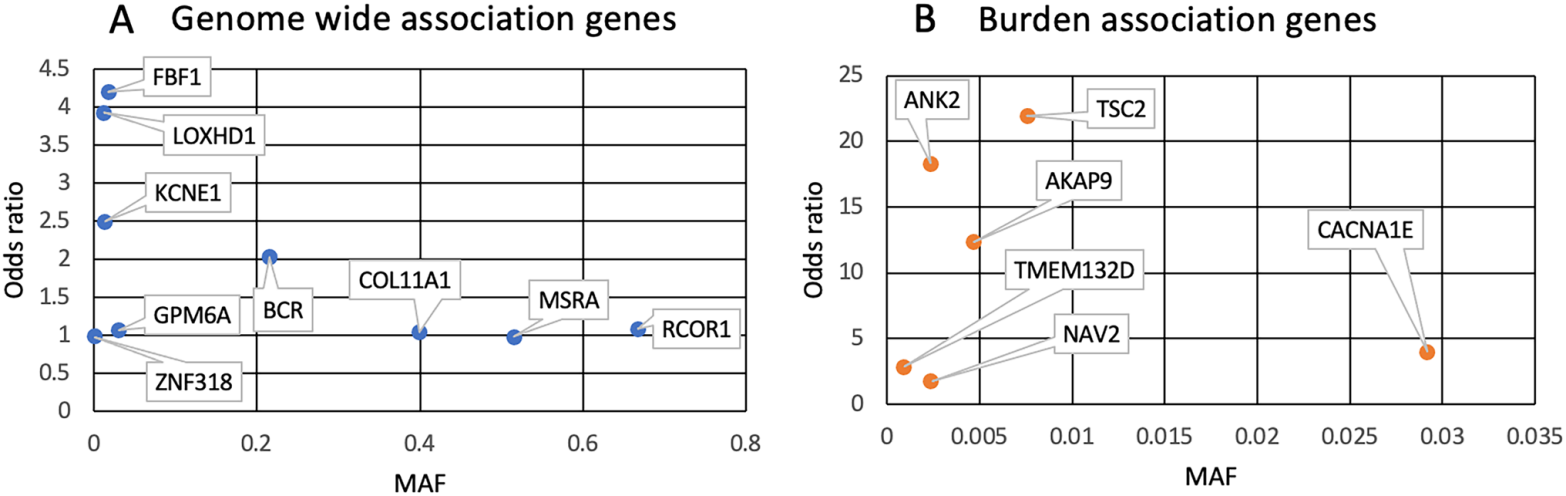
The genetic landscape of tinnitus. The effect of the variants associated with tinnitus as a function of their allelic frequency was observed in tinnitus genomic studies. **A** Blue dots represent OR for common variants associated with self-reported tinnitus in genome-wide association studies (GWAS). Odds ratios from GWAS studies were calculated from beta values from their respective studies. Some studies have not been included since information on neither beta nor odds ratio was published. **B** *Orange dots* represent OR for genes enriched in rare missense variants in exome/genome sequencing studies selecting an individual with tinnitus extreme phenotype or specific population cohorts through burden test analysis. Odds ratios represent the entire gene enrichment, not a single variant. However, the MAF value represents the MAF of the most common variant reported in the analysis. If variants were not reported, the higher MAF values were used

All of them sought to find genetic variants associated with the development of tinnitus (Table 2 and Supplementary information). Figure 2 summarizes the main genes reported in GWAS and gene burden analyses. None of the candidate gene studies was replicated.

**Table 2:** Summary of the main findings in the genetic association studies obtained

**Reference**	**Country**	**Design**	**Genes**	**Main objective**	**Sample size**	**Sex (male/female)**	**Average age (years)**	**Tinnitus prevalence**	**SNHL prevalence**	**Results**	**Replication**
Amanat et al. [8]	Spain	WES	*ANK2*, *TSC2*, *AKAP9*	To identify rare variants in synaptic genes by exome sequencing in patients with severe tinnitus	*n* = 91; cases: 59; controls: 32	Cases: 42 M–17F; controls: unknown; replication cohort (cases: 42 M–55F; controls: unknown)	N/A	Cases: 100% (persistent tinnitus); controls: 0% (fluctuating tinnitus)	Reference cohort: N/A Replication cohort: 64%	Enrichment of rare missense variants in 24 synaptic genes in a Spanish cohort (more significant in *PRUNE2, AKAP9, SORBS1, ITGAX, ANK2, KIF20B* and *TSC2* (*p* < 2E − 04)), compared with reference datasets. It was replicated for *ANK2* in a Swedish cohort, and in a subset of 34 Swedish patients with severe tinnitus for *ANK2, AKAP9* and *TSC2* (*p* < 2E − 02). This association was not significant in a third cohort of 701 generalized epilepsy individuals without tinnitus	Yes Independent WGS Swedish cohort (TIGER) *n* = 97 Cases: 34; controls: 63 Independent epilepsy WES cohort (CoGIE) *n* = 701 Cases: 152 Control: 549
Bhatt et al. [48]	USA	Panel	*KCNQ1, KCNE1*	To examine the relationship between selected genetic variants and measures of tinnitus in a sample of young musicians	*n* = 186; cases: 106; controls: 80	Cohort: 99 M–87F	20.3	Cases: 100%; controls: 0%	N/A	Individuals with at least one minor allele of rs163171 (C > T) in *KCNQ1* exhibit significantly higher odds of reporting tinnitus compared to individuals carrying the major allele of rs163171. *KCNE1* rs2070358 revealed a suggestive association (*p* = 0.049) with tinnitus, but the FDR corrected *p*-value did not achieve statistical significance (*p* < 0.05)	No
Bhatt et al. [9]	UK	GWAS	*GPM6A*	To conduct a GWAS analysis in the UK Biobank, adjusting for known environmental risk factors, and interrogating the genetic underpinnings of tinnitus-related distress	*n* = 132,438; cases: 38,525; controls: 93,013	Cohort: 61,646 M–70,792F	40– > 70	Cases: 100%; controls: 0%	N/A	A genomic region containing SNP (rs71595470) near *GPM6A* revealed a significant association with tinnitus, and 19 SNPs showed suggestive associations with tinnitus. Fifteen SNPs showed association with tinnitus-related distress. The enrichment analysis with FUMA identified 23 gene sets associated with tinnitus	No
Gallego-Martinez et al. [49]	Sweden	WGS	*CACNA1E, NAV2, TMEM132D*	To explore the association of rare single-nucleotide variants (SNVs), large structural variations (LSVs), and copy number variants (CNVs) in the genome of Swedish patients with severe tinnitus	*n* = 97 (TIGER cohort)	TIGER cohort: 43 M–54F; Replication cohort: 146 M–152F	46 ± 12.86 (TIGER cohort) 47 ± 11.52 (replication cohort)	100% (TIGER cohort) 72.2% (replication cohort)	64% (TIGER cohort; 79% in severe tinnitus cohort) 56% (replication cohort)	Enrichment of rare missense variants in 6 and 8 high-constraint genes in SEVTIN and TIGER cohorts, respectively. There is also an enrichment of missense variants in the *CACNA1E* in both SEVTIN and TIGER. The burden of missense variants was replicated in 9 high-constrained genes in the JAGUAR cohort, including the *NAV2*, compared with NFE. Moreover, LSVs in constrained regions overlapping *CACNA1E*, *NAV2*, and *TMEM132D* were observed in TIGER and SEVTIN	Yes Independent Swedish WES cohort (JAGUAR) *n* = 298; cases: 143; control: 155
Haider et al. [35]	Portugal	Genotyping Cohort vs reference population	*GRM7*	To study the relationships between presbycusis, tinnitus, co-morbidities, and the genotypes of *GRM7* and *NAT2*, in a sample of older Portuguese adults	*n* = 78	33 M–45F	64.6 (± 5.58)	64.1%, 24% (severe tinnitus)	24%	For *GRM7* gen, individuals with a T/T genotype have a higher risk for age-related HL and 33% lower risk for tinnitus, compared to individuals with A/A and A/T genotype, respectively. Allele AT of *GRM7* can have a statistically significant influence toward the severity of tinnitus	No
Jeong et al. [36]	South Korea	Genotyping Case–control study	*BDNF, HTTLPR*	To investigate the association of *BDNF* Val66Met or *5-HTTLPR* polymorphisms with tinnitus and the mediating effects of psychological distress	*n* = 338 Cases: 86; controls: 252	Cases: 41 M–45F; controls: 132 M–120F	Cases: 53.5 ± 13.7; controls: 53.1 ± 10.4	Cases: 100%; controls: 0%	SNHL patients were excluded	No association were found between groups regarding *BDNF* Val66Met (*p* = 0.142) and 5-*HTTLPR* (*p* = 0.054). The mean THI score was significantly higher in patients with the s/s genotype (47.9 (18.9)) than in those with l/s or l/l genotype (38.2 (25.5)) of 5-*HTTLPR* (*p* = 0.024)	No
Lechowicz et al. [37]	Poland	MiDNA sequencing Case–control study	Mitochondrial genes	To investigate the prevalence of tinnitus among Polish HL patients with identified pathogenic mtDNA variants	*n* = 114; cases: 17; controls: 97	Cases: 7 M–10F; controls: N/A	N/A	Cases: 17.6%; control: 100%	Cases: 100%; controls: 69%	There were no statistically significant differences in the prevalence of tinnitus between HL patients with mtDNA variants and the general Polish population. There were no statistically significant differences in tinnitus annoyance (VAS-A) between al the subgroups of tinnitus patients. In Polish HL patients with tinnitus, m.7511 T > C was significantly more frequent than in patients without tinnitus (*p* = 0.0441)	No
Orenay-Boyacioglu et al. [38]	Turkey	Genotyping case–control study	*GDNF*	To investigate the role of *GDNF* polymorphisms in tinnitus pathophysiology	*n* = 94; cases: 52; controls: 42	Cases: 33 M–19F; controls: 29 M–13F	Cases: 43.6 ± 10.7; controls: 39.3 ± 9.8	Cases: 100%; controls: 0%	N/A	No association was found for rs884344 and rs3812047 and subjects with tinnitus. Heterozygosity was significantly lower for *GDNF* rs1110149 polymorphism in tinnitus subjects compared to the controls (*p* = 0.02)	No
Orenay-Boyacioglu et al. [44]		WGBS specific regions	*BDNF*, *GDNF*	To study the relationship between the promotor methylation of BDNF and GDNF genes and chronic tinnitus in peripheral blood samples	*n* = 110; cases: 60; controls: 50	Cases: 39 M–21F; controls: 31 M–19F	Cases: 36.5 (21–52); controls: 38.5 (23–54)	Cases: 100%; controls: 0%	N/A	Statistically significant differences were detected between *BDNF* CpG6 and *GDNF* CpG3-5–6 methylation ratios in the comparison of control group and the chronic tinnitus patients (*p* = 0.002, 0.0005, 0.00003, and 0.0029, respectively)	No
Rottenberg et al. [45]	Slovenia	Genotyping case–control study	*GABA(A*) beta-3 subunit gene	To explore associations between manifestation of tinnitus, auditory evoked potentials and genetic background of gamma-aminobutyric acid type A (GABA(A) receptors) to support the disinhibited feedback hypothesis of tinnitus generation	*n* = 131	Cohort: 61 M–71F	Cohort: 52 ± 13.8	Cohort: 100%	Cohort: 100%	Statistically significant difference in the tinnitus score in relation to the genotype of (CA)n tandem repeat of the GABRß3 receptor subunit gene (*p* = 0.002)	No
Urbanek et al. [47]	UK	GWAS	*WDPCP*	To define underlying genes that may preclude tinnitus, through a GWAS in the UK Biobank	*n* = 23,742; cases: 526; control: 19,047	Cases: 226M300F; controls: 7315 M–11,732F	40–70	Cases: 100%; controls: 0%	Self-reported SNHL patients were excluded	Seventeen suggestive SNP (*p* < 1e − 5) spanning 13 genes were identified in two sex-separated cohorts reporting chronic, bothersome tinnitus. A significant missense mutation in *WDPCP* (*p* = 3.959e − 10) was identified in the female cohort	No
Vanneste et al. [39]	USA	Genotyping case–control study	*COMT*	To study the role of *COMT* polymorphisms in the activity in the ventromedial PFC/anterior cingulate cortex and its effect on tinnitus perception	*n* = 60; cases: 40; controls: 20	Cases: 28 M–12F; controls: 13 M–7F	Cases: 45.97 ± 14.19; controls: 45.6 ± 16.27	Cases: 100%; controls: 0%	No significant differences in audiograms between healthy controls and tinnitus patients	An interaction between the SNHL degree and the *COMT* Val^158^Met polymorphism can increase susceptibility to the clinical manifestation of tinnitus (loudness). No significant was observed in THI scores between the Val/Val genotype and Met carriers	No
Vanneste et al. [40]	USA	Genotyping case–control study	*BDNF*	To study the effect of *BDNF* Val/Met carriers and Val-homozygotes and perception of distress due to tinnitus	*n* = 110; cases: 55; controls: 55	Cases: 38 M–17F; controls: 36 M–19F	Cases: 54.49 ± 15.3; controls: 54.63 ± 12.7	Cases: 100%; controls: 0%	No significant differences in audiograms between healthy controls and tinnitus patients	Val/Met carriers have a higher stress level in comparison to Val homozygotes (controls: *F*(1,106) = 66.97, *p* < 0.001; cases: *F*(1,106) = 54.68, *p* < 0.001). For the tinnitus group, we further show that there is a significant effect between Val homozygotes and Val/Met carriers for tinnitus-related distress (*F*(1,53) = 8.45, *p* = 0.005)	No
Watabe et al. [43]	Japan	Genotyping case–control study	*BCR*	To study the association between the grade of tinnitus distress and the genetic background, to identify prognostic markers	*n* = 138	Cohort: 59 M–79F	Cohort: 61.3 ± 13.1	Cohort: 100%	N/A	rs131702 of *BCR* is independent of depression in this study and is, therefore, a prognostic factor unique to tinnitus	No
Wells et al. [46]	UK	GWAS	*RCOR1*	To conduct a GWAS analysis using self-reported tinnitus in the UK Biobank	*n* = 134,429; cases: 14,829; controls: 119,600	Not defined	N/A	Cases: 100%; controls: 0%	N/A	Three variants in close proximity to the *RCOR1* gene reached genome wide significance: rs4906228 (*p* = 1.7E − 08), rs4900545 (*p* = 1.8E − 08) and 14:103042287_CT_C (*p* = 3.50E − 08)	No
Xie et al. [41]	China	GWAS	*TNFRSF1A*	To identify novel loci related to the risk of noise-induced tinnitus in the Chinese population	*n* = 298; cases: 65; controls: 233	100% males	Cases: 23.8 (1.6 SD); controls: 23.4 (1.6 SD) cases: 26.4 (3.9 SD)* controls: 24.5 (2.9 SD)*	Cases: 100%; controls: 0%	Cases: 100%; controls: 0% (noise exposure 100% subjects)	Two SNVs: rs2846071 (OR = 2.14 (1.96–3.4), combined *p* = 4.9 × 10^−6^); rs4149577 in the intron of *TNFRSF1A* gene at 12p13.31 (OR = 2.05 (1.9–2.51), combined *p* = 6.9 × 10^−6^), are associated to noise-induced tinnitus	Yes Independent case and control cohort *n* = 413 Cases: 34; control: 379
Yüce et al. [42]	Turkey	Genotyping case–control study	*ACE; ADD1*	To investigate the relationship between severe chronic tinnitus and angiotensin-converting enzyme (ACE) I/D and α-adducin (ADD1) *G460W* gene polymorphisms	*n* = 193; cases: 89; control: 104	Cases: 41 M–48F; controls: 54 M–50F	Cases: 48.1 ± 13.5; controls: 45 ± 16	Cases: 100% (severe tinnitus); controls: 0%	SNHL patients were excluded	Combined genotype frequencies for both *ACE* and *ADD1* allelic variants were higher in the patient group than in the control group (*p* = 0.007)	No

*NFE* non-Finnish European, *(SN)HL* (sensorineural) hearing loss

The allelic variants and genes reported were associated with the following mechanisms:

- **Regulation of the neural activity**, including the auditory pathway, such as the *BDNF* (brain-derived neurotrophic factor) gene and the *GDNF* (glial cell line-derived neurotrophic factor) gene, both involved in the early development of central auditory pathway and the inner ear; the *ANK2* (Ankyrin 2) gene, which encodes two different polypeptide including giant Ankyrin-2, a neuro-specific isoform variant expressed broadly in the central nervous system that keeps connectivity and neural activity in the CNS; the *GPM6A* (glycoprotein M6A) gene, which encodes neural glycoprotein M6a and plays an essential role in neural growth; the *NAV2* (neuron navigator 2) gene, which is involved in neuronal and different sensory organs development; the *TMEM132D* (transmembrane protein 132D) gene, which encodes a transmembrane protein known for its capacity to act as a cell-surface marker for oligodendrocyte differentiation and neuronal morphogenesis; *BCR* (breakpoint cluster region) gene, which encodes the Rho family low molecular weight G-protein, abundantly expressed in the central nervous system and crucial for neurogenesis; and *RCOR1* (REST corepressor 1) gene which encodes a component of a transcriptional repressor complex which represses neuronal gene expression in non-neuronal cells.
- **Receptors or transporters of neurotransmitters**, including the polymorphic region (5-HTTLPR) of the serotonin transporter gene SLC6A4; the *GRM7* gene, which encodes the metabotropic glutamate receptor subtype 7 (mGluR7); and the *GABRB3* (GABA(A) beta-3 subunit) gene, which encodes a receptor for neuromediators involved in the descending part of the auditory pathways.
- **Metabolism and enzymatic pathways**, such as the *AKAP9* (A-kinase anchoring protein 9) gene, whose known function is binding to the protein kinase A (PKA) regulatory subunit to enclose it to different parts of the cell where phosphorylation is needed; the *COMT* (catechol-O-methyltransferase) gene, which inactivates dopamine, norepinephrine, and epinephrine neurotransmitters in the mammalian brain; and the *ACE* (angiotensin-converting enzyme) gene, which encodes a crucial enzyme in the renin-angiotensin system and is related to the cardiovascular and body water regulation.
- **Voltage-gated channels and cellular homeostasis mediators**, such as the *TSC2* (tuberous sclerosis complex 2) gene, which encodes a tumor suppressor protein part of the TSC involved in the negative regulation of mTORC1 (mechanistic target of rapamycin complex 1) activity; the *CACNA1E* (calcium voltage-gated channel subunit alpha1 subunit E) gene, which encodes a part of the “high-voltage activated” channel involved in the firing patterns modulation of neurons important for information processing, the *ADD1* (α-adducin) gene, which is related to the volume and sodium homeostasis by interacting with the epithelial sodium channel; the *KCNQ1* (potassium voltage-gated channel subfamily Q member 1); and *KCNE1* (potassium voltage-gated channel subfamily E regulatory subunit 1), which form a voltage-gated potassium channel expressed in the marginal cell membrane of the stria vascularis.
- **Inflammation**, such as the *TNFRSF1A* (tumor necrosis factor receptor superfamily member 1A) gene, which encodes a member of the TNF receptor superfamily of proteins involved in the TNF pathway. Two non-coding variants showed an association and were replicated in the Chinese population associated with noise-induced tinnitus (rs2846071; rs4149577).
- **Structural genes**, such as *WDPCP* (WD repeat containing planar cell polarity) gene, which is related to the PCP effector proteins to regulate ciliogenesis during development and regulation of the actin cytoskeleton.
- **Mitochondrial DNA variants related to hearing loss.**

Although four studies replicated their findings in another cohort, with similar characteristics to the discovery cohort [8, 9, 41, 49], none of the other studies replicated their findings in a second independent cohort.

### Quality Assessment of Studies

The detailed analysis based on the seven domains of ROBINS-E is summarized in Table 3. According to this, fifteen of the studies had a moderate risk of bias [8, 22, 24–27, 29, 30, 35–37, 39–42], and twelve studies were evaluated to have a high risk of bias [4, 23, 28, 31–34, 38, 43–45, 48].

**Table 3:**
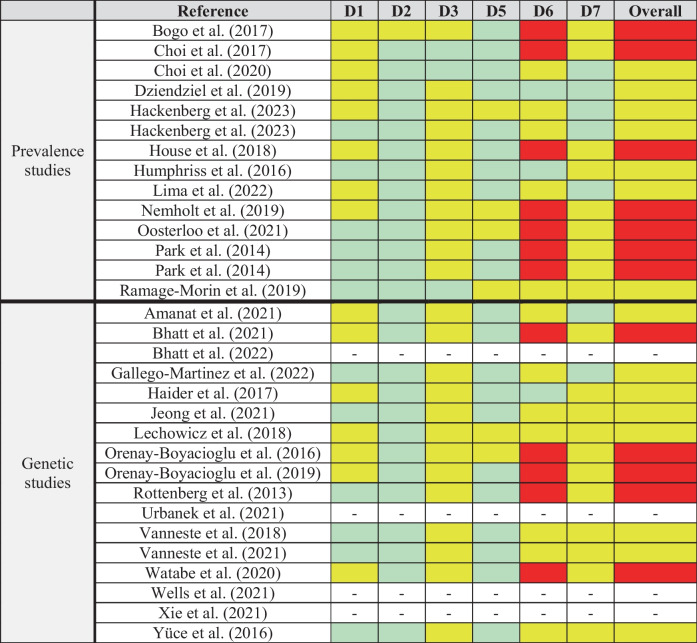
Risk of bias assessment according to the risk of bias in non-randomized studies of exposures (ROBINS-E) tool. GWAS studies were excluded from this analysis*

*No specific tool is available for such aim

## Discussion

Tinnitus is a multifactorial condition and a growing health problem associated with hearing loss. It is related to a wide variety of comorbidities, such as anxiety, hyperacusis, SNHL, headache and some otologic and neurologic conditions [50]. Moreover, tinnitus has a significant impact on the lives of patients who suffer from it and represents an economic burden for the health system [51].

Tinnitus has a significant heritability according to twins [3, 4], adoptees [5], and familial aggregation studies [6]; however, tinnitus heterogeneity and its association with several comorbidities make it difficult to decipher the genetic variation contributing to tinnitus. This challenge is higher with common variants located in non-coding regions that may show small effect by regulating multiple genes in different pathways. In this work, we have reviewed the records published in the last 10 years, on the genetic basis of tinnitus, after establishing a search strategy focused on prevalence studies to compare differences across different populations, genetic association studies, and familial aggregation studies.

One of the novel approaches of this systematic review is to consider the evaluation of the hearing as an essential part of the tinnitus phenotyping, as both conditions have a strong association [52]. For this reason, we have limited the selection to genetic studies that included the hearing thresholds of participants to control the confounding effect of genetic variants associated with hearing loss in tinnitus [53]. This has led to the exclusion of some GWAS reporting genetic associations with tinnitus; however, these genetic findings should be considered with caution, since these studies did not include hearing loss data and/or included military veterans with a history of noise exposure as replication cohort [8, 11, 49].

Several GWAS using biobank datasets have reported few common variants with significant associations with tinnitus [9, 11, 46, 47]; however, these studies were based on the participant responses to general health questionaries where tinnitus was self-reported, without a confirmed diagnosis in digital health records. Furthermore, these studies did not include the hearing thresholds, and it is difficult to determine if the reported associations are mediated by the underlying hearing loss, which is associated with tinnitus in the general population, particularly those over 50 years old. Finally, most of these studies did not include an independent replication cohort, and these associations have not been confirmed in later studies using other UK Biobank datasets [46, 47].

### Prevalence and Tinnitus Phenotype

Tinnitus can receive different definitions, but there are some major characteristics, like its duration, that help to define this symptom. According to the latest clinical practice guidelines [54], chronic tinnitus is defined by a duration of at least 6 months. Most of the genetic studies present a homogeneous distribution of tinnitus patients, based on the duration criteria for chronic tinnitus. However, this information could be missed in some prevalence studies based on self-reported questionnaires [55].

Tinnitus disorder is a condition with a lower frequency in the general population. A more precise definition of tinnitus is needed for clinical and genomic research studies.

A standard approach to investigate the combined effect of environmental and genetic contributions in complex disorders is to compare prevalence across different populations with different ancestry living in the same geographical area or a specific population migrating to another continent. Here, we have compared the prevalence of tinnitus to determine the effect of ethnicity and population structure on tinnitus. The genetic uniformity, based on the reproduction between similar individuals or subjects with common ancestors, results in a decrease in the genetic diversity with a lower allele enrichment and less responsiveness to environmental changes [7].

Most studies report conservative tinnitus prevalence rates to be between 10 and 19% of adults [52, 56]. In addition, annoying tinnitus usually affects a low percentage of these patients [57]. Our results show slightly higher rates for both tinnitus prevalence (26.3%) and bothersome tinnitus (20%), which could be partly explained by the approach of the questions regarding tinnitus, in a self-reported questionnaire. Of note, the study of Choi et al. [22] performed in the USA reported a prevalence three times higher in white European compared to the Asian population and intermediate values for Hispanic and African-American individuals. Further prevalence studies in ethnically diverse countries are needed to compare tinnitus incidence in the same geographical areas according to ancestry.

### Genetic Signature of Tinnitus

Some recent studies have integrated genetic knowledge into the tinnitus background, using different techniques. The main approaches focus on single variation and association analysis. GWAS have identified and replicated common variants in patients self-reporting tinnitus and noise-induced hearing loss, as well as tinnitus related to misophonia in population-based cohorts [9, 11, 41, 46, 47, 58]. GWAS studies have been useful in targeting potential regulators in tinnitus development in large population cohorts. UK Biobank has been demonstrated to be a valuable resource for association analysis using both self-reported questionnaires for tinnitus and genetic data. Different studies using this cohort have successfully pinpointed potential biomarkers for tinnitus; however, most of these studies lack a replication cohort to conclusively identify the causal genes, and these markers are common variants with a small effect [59, 60]. Of note, Trpchevska et al. [61] performed a large tinnitus GWAS including 723,166 participants from different cohorts, but no signal reached GWAS significance.

An alternative approach consists of selecting individuals with extreme phenotype (individuals with severe or mild symptoms at the ends of the phenotype distribution) and using omics data to identify rare and ultrarare variants by gene burden analysis [62]. This approach leads to more accurate identification of candidate genes. An enrichment of rare variants in patients with severe tinnitus has allowed the identification of target genes, which were replicated in an independent cohort [8]. Burden tests for rare biomarkers have been used successfully to identify potential genes with different rare variants enriched in tinnitus cohorts, using both exome and genome data [8, 49]. However, this overload of rare variation in certain genes may be a population-specific effect, and functional analyses of these rare variants are needed using cellular and animal models. Population stratification can be reasonably ruled out by segregation analysis of rare variants in multiple unrelated individuals.

A third approach is the combination of multiple bioinformatic tools analyzing different types of rare variants (i.e., single nucleotide variants, short indels, large structural variants, or copy number variants), as it has been described in other brain disorders [63, 64]. The identification of genes such as *CACNA1E* or *NAV2*, showing enrichment of missense and large structural variants in patients with tinnitus may lead to defining new druggable targets for tinnitus. However, this approach is limited by the clinical information of the cohort, in order to control the effect of other associated comorbidities.

A better understanding of the genetic structure of tinnitus may lead to explaining the difference in the phenotype. Though tinnitus may be a common symptom, tinnitus disorder, in its current definition as a condition associated with emotional, cognitive, or behavioral changes, may be considered a rare disease (less than 1–2% of the general population compared with tinnitus as a symptom). The differences between both may be the result of the combined effect of multiple common and rare variants, with an additive or epistatic effect leading to a complete or severe phenotype.

## Limitations

This systematic review has some limitations. As most of the prevalence studies were performed by retrieving data from population-based registries, the information available for tinnitus phenotyping, including its time of evolution, laterality, or psychoacoustic characteristics, is incomplete. Tinnitus is a heterogeneous symptom, so it is essential to perform a deep phenotyping of this condition, including all the reported comorbidities to control association biases.

A second limitation is the low sample size associated with the tinnitus extreme phenotype approach that limits statistical power and cannot avoid population-specific effects. However, the main concern for most of the genetic studies is the lack of a second independent cohort to replicate genetic associations.

Since most of the reviewed studies exhibited a moderate to high risk of bias, the conclusions must be considered with caution, and future genetic studies should include a more precise selection of tinnitus individuals and a validation cohort.

## Conclusions

1. The genetic contribution to tinnitus is mediated by common and rare variations, and it is likely to have population-specific effects.
2. Common allelic variants associated with tinnitus with a small effect have been associated with noise-induced tinnitus.
3. Rare missense variants with a large effect have been associated with severe tinnitus, although their effect on other comorbidities such as hearing or hyperacusis has not been established.

### Supplementary Information

Below is the link to the electronic supplementary material.

Supplementary file1 (XLSX 17 KB)

Supplementary file2 (DOCX 132 KB)
