# Supplementary material for: A Systematic Review on the Genetic Contribution to Tinnitus
Source: J Assoc Res Otolaryngol. 2024 Feb 9;25(1):13–33. doi: 10.1007/s10162-024-00925-6 (PMC10907330; doi:10.1007/s10162-024-00925-6)
Supplement: Supplementary file 2 — Supplementary file2 (DOCX 132 KB) [file 10162_2024_925_MOESM2_ESM.docx]

**Annex 1:** PRISMA 2020 checklist of the systematic review.

| **Section/topic** | **#** | **Item** | **Figure p.#** |
| --- | --- | --- | --- |
| **TITLE** |  |  |  |
| Title | 1 | Identify the publication as a systematic review, meta-analysis or both. | 1 |
| **ABSTRACT** |  |  |  |
| Structured summary | 2 | Provide a structured summary including, as appropriate: background; objectives; source of data; eligibility criteria of studies, participants, and interventions; assessment of studies and methods of synthesis; results; limitations; conclusions and implications of main findings; registration number of the systematic review. | 2 |
| **INTRODUCTION** |  |  |  |
| Justification | 3 | Describe the rationale for the review in the context of what is already known on the subject. | 3 |
| Objectives | 4 | Explicitly state the questions you want to answer in relation to participants, interventions, comparisons, outcomes and study design (PICOS). | 3 |
| **METHODS** |  |  |  |
| Protocol and registration | 5 | Indicate whether there is a review protocol that can be accessed (e.g. web address) and, if available, information on the registry, including its registration number. | 4 |
| Eligibility criteria | 6 | Specify the characteristics of the studies (e.g. PICOS, duration of follow-up) and characteristics (e.g. years covered, languages or publication status) used as eligibility criteria and their justification. | 4 |
| Sources of information | 7 | Describe all sources of information (e.g. databases and search periods, contact with authors to identify additional studies, etc.) in the search and the date of the last search performed. | 4 |
| Search | 8 | Present the complete electronic search strategy in at least one database, including the limits used in such a way that it can be reproduced. | 4 |
| Selection of studies | 9 | Specify the study selection process (e.g. screening and eligibility included in the systematic review and, where relevant, included in the meta-analysis). | 4 |
| Data collection process | 10 | Describe the methods for extracting data from publications (e.g. directed, duplicate and independent forms) and any processes for obtaining and confirming data by researchers. | 4 |
| List of data | 11 | List and define all variables for which data were sought (e.g. PICOS source of funding) and any assumptions and simplifications made. | 4 |
| Risk of bias in individual studies | 12 | Describe the methods used to assess the risk of bias in individual studies (specify whether this was done at the study or outcome level) and how this information has been used in the data synthesis. | 5 |
| Summary measures | 13 | Specify the main summary measures (e.g. risk ratio or mean difference). | 4 |
| Summary of results | 14 | Describe methods for handling data and combining study results, if done, including measures of consistency (e.g. quantification of heterogeneity using the I-statistic index^2^ ) for each meta-analysis. | 5 |
| Risk of bias between studies | 15 | Specify any assessment of risk of bias that may affect the cumulative evidence (e.g. publication bias or selective reporting). | 5 |
| Further analysis | 16 | Describe additional methods of analysis (e.g. sensitivity or subgroup analysis, meta-regression), if done, indicate which were pre-specified. | N/A |
| **RESULTS** |  |  |  |
| Selection of studies | 17 | Provide the number of studies screened, assessed for eligibility and included in the review, and detail the reasons for exclusion at each stage, ideally by means of a flow chart. | 6, Figure 1 |
| Characteristics of the studies | 18 | For each study, present the characteristics for which data were extracted (e.g. size, PICOS and duration of follow-up) and provide bibliographic citations. | 6, 7, 8, Tables 1-2 |
| Risk of bias in studies | 19 | Present data on the risk of bias in each study and, if available, any assessment of bias in the results (see item 12). | 8, Table 3 |
| Results of individual studies | 20 | For each outcome considered for each study (benefits or harms), present (a) the summary data for each intervention group and (b) the effect estimate with its confidence interval, ideally in graphical form using a forest plot.) | N/A |
| Summary of results | 21 | Present results of all meta-analyses performed, including confidence intervals and measures of consistency. | N/A |
| Risk of bias between studies | 22 | Present the results of any assessment of risk of bias between studies (see item 15). | 8, Table 3 |
| Further analysis | 23 | Provide the results of any additional analyses, if conducted (e.g. sensitivity or subgroup analyses, meta-regression (see item 16)). | 6, 7, 8, Table 1-2 |
| **DISCUSSION** |  |  |  |
| Summary of evidence | 24 | Summarize the main findings, including the strength of the evidence for each main outcome; consider its relevance to key groups (e.g. care providers, users and health decision-makers). | 9-11 |
| Limitations | 25 | Discuss limitations of the studies and results (e.g. risk of bias) and of the review (e.g. incomplete collection of identified studies or selective reporting). | 11 |
| Conclusions | 26 | Provide a general interpretation of the results in the context of other evidence as well as implications for future research. | 12 |
| **FINANCING** |  |  |  |
| Funding | 27 | Describe the sources of funding for the systematic review and other support (e.g. data contribution), as well as the role of funders in the systematic review. | 12 |

N/A: not applicable
